# Wound healing of metastatic perineal Crohn’s disease using hyperbaric oxygen therapy: A case series

**DOI:** 10.1177/2050640620934915

**Published:** 2020-06-12

**Authors:** Corine A Lansdorp, Christianne J Buskens, Krisztina B Gecse, Geert RAM D’Haens, Rob A Van Hulst

**Affiliations:** 1 Department of Anaesthesiology/Hyperbaric Medicine, Amsterdam University Medical Centre, Location AMC, Amsterdam, The Netherlands; 2 Department of Surgery, Amsterdam University Medical Centre, Location AMC, Amsterdam, The Netherlands; 3 Department of Gastroenterology and Hepatology, Amsterdam University Medical Centre, Location AMC, Amsterdam, The Netherlands

**Keywords:** Inflammatory bowel disease, cutaneous manifestations, wound healing

## Abstract

**Background:**

Metastatic Crohn’s disease (CD) is a rare manifestation of CD. It involves inflammatory skin lesions with histopathological findings (granulomas) similar to CD, without connection to the gastrointestinal tract. Hyperbaric oxygen therapy (HBO) has been suggested as a possible treatment option.

**Objective:**

This study aimed to identify and treat a consecutive series of patients with biopsy-proven metastatic CD and monitor wound healing using prospectively acquired outcomes.

**Methods:**

Pathology results of all patients with ongoing perineal wound-healing problems after proctectomy between 2005 and 2018 at the Amsterdam University Medical Centre were assessed for metastatic CD. Patients with a biopsy-proven diagnosis of perineal metastatic CD were offered HBO (40 daily sessions of 100% oxygen at 2.4 atmosphere absolute). Wound healing was monitored using photographs and standardised questionnaires (the Inflammatory Bowel Disease Questionnaire, EuroQol Visual Analogue Scale and the Female Sexual Function Index) at baseline and 1 and 3 months after HBO.

**Results:**

Out of 13 patients in the cohort with persisting perineal wounds after proctectomy, six (46%) had biopsy results consistent with metastatic CD. Of these, three accepted treatment with HBO. All three patients were female. One patient had complete healing of her perineal wound; another patient showed initial improvement but had a flare of luminal and perineal disease at the 3-month follow-up. The third patient showed improvement solely in the questionnaires, with higher scores on all three questionnaires.

**Conclusion:**

A high rate of metastatic CD was found in patients with ongoing wound-healing problems after proctectomy, implying that the disease might not be as rare in these selected patients as previously thought. HBO might be beneficial in the treatment of metastatic CD.

## Introduction

Crohn’s disease (CD) is a chronic inflammation of the gastrointestinal (GI) tract, which is also frequently associated with extra-intestinal manifestations.^1^ Metastatic CD refers to a rare manifestation (reported in as few as 100 cases to date) involving inflammatory lesions of the skin, with histopathological findings (epithelioid granulomas) similar to CD.^1–4^ It most often affects the extremities or intertriginous skin, but the face and genitalia can also be involved. By definition, these lesions are separated from the GI tract by normal tissue, thereby excluding classic perianal CD. Diagnosis can be difficult, as the clinical features of perineal metastatic CD can differ greatly, and distinction from other diseases such as hidradenitis suppurativa can usually only occur based on biopsy results (granulomas not present in hidradenitis suppurativa). Metastatic CD does not necessarily mirror the activity of CD in the intestine.^5^ Treatment of these patients is challenging. Options that have been proposed in literature include metronidazole, azathioprine with topical steroids and infliximab, but such treatments have only been reported in single case reports. Treatment also frequently involves surgical approaches such as cleaning of wounds and vacuum therapy, or surgical resection of the (meso)rectum. Previously, it has been suggested that hyperbaric oxygen therapy (HBO) may provide a beneficial effect on metastatic CD.^1,2,6^

HBO consists of breathing 100% oxygen for 60–90 minutes under a higher than normal atmospheric pressure: usually 2.0–2.5 atmosphere absolute (ATA). This increases plasma and tissue oxygen levels, and decreases hypoxia. It has been shown to alter several signalling pathways involved in tissue response to hypoxia and wound repair, and suppresses the production of pro-inflammatory cytokines.^7,8^ Also, in general, oxidative stress is recognised to play a role in stem-cell mobilisation and promote wound healing.^9^ The Undersea and Hyperbaric Medical Society, a non-profit organisation that plays an important role in providing scientific and medical information on hyperbaric medicine, lists 14 indications for HBO therapy.^10^ These include late radiation tissue injuries, diabetic foot ulcers and carbon monoxide poisoning. Treatment for chronic problems (e.g. wound healing) usually involves giving daily sessions for 6–8 weeks. The therapy is generally considered safe with few complications, with barotrauma of the ears or sinuses and transient myopia being the most common.^11^

HBO has been reported as a potential adjunctive treatment in patients suffering from inflammatory bowel disease (IBD).^8,12^ However, there has been only one case report investigating the effect of HBO specifically for metastatic CD, which showed a positive outcome.^6^

In this case series, we present the outcomes of three patients with metastatic perineal CD who were treated with HBO.

## Methods

Medical records of all adult patients who underwent intersphincteric proctocolectomy or completion proctectomy for CD between January 2005 and January 2018 at the Amsterdam University Medical Centre (UMC) were retrospectively assessed for ongoing wound-healing problems at least 1 year after surgery. In case of ongoing perineal wounds, available official pathology results of perineal tissue biopsies were assessed retrospectively for a diagnosis of metastatic CD, defined as the presence of granulomas consistent with CD. All patients with a proven diagnosis of cutaneous CD were offered HBO as an experimental treatment option after a multidisciplinary discussion. If patients accepted HBO treatment, written consent for the collection of data concerning their medical history and treatment with HBO was obtained.

All patients had an examination to determine fitness for HBO before treatment. They were treated with 40 daily sessions (excluding weekends) of 80 minutes of 100% oxygen at 2.4 ATA. Wound healing was documented by photographs, and standardised questionnaires were used to evaluate quality of life. These questionnaires were:

- Inflammatory Bowel Disease Questionnaire (IBDQ), with scores ranging from 32 to 224, where higher scores indicate a better quality of life. A relevant clinical response has been defined as an increase of ≥27 points, and remission as a score of ≥168.^13^
- EuroQol Visual Analogue Scale (EQ-VAS) that records the respondent’s self-rated health on a scale from 0 to 100, with higher scores indicating a higher quality of life.
- Female Sexual Function Index (FSFI), with scores ranging from 2 to 32, where higher scores indicate higher sexual functioning. A score >26 reflects normal sexual function.^14^

Wound healing and quality of life were assessed at baseline and 1 and 3 months after HBO.

## Results

Between January 2005 and January 2018, 64 patients underwent proctectomy for CD. Of these patients, 44 had perianal fistulas as (one of) the indication(s) for their surgery. Out of the 64 patients, 13 had ongoing perineal wounds for more than 1 year after proctectomy (all of which had perianal fistulas at the time of surgery). Assessment of available pathology results showed that 6/13 (46%) had biopsy results consistent with metastatic CD. Furthermore, one patient had an accumulation of multiple multinucleate giant cells, possibly forming a granuloma, but due to cauterisation effects of the tissue, a definite diagnosis could not be made. Three patients had biopsy reports without the specific mention of granulomas, and three patients did not have a biopsy of their perineal wounds performed in the past.

Out of the six patients who were identified with a proven diagnosis of metastatic CD, five were offered HBO. The other patient was not eligible for HBO due to ongoing pregnancy. Two patients refused because they had little complaints of their wounds, and three patients accepted.

All patients were female, with therapy-refractory perineal wounds after proctectomy and mesorectal excision with omentoplasty to fill the defect. All of these patients had a concomitant diagnosis of hidradenitis suppurativa. All patients had been treated for years with possible conservative treatment, percutaneous drainage and surgical intervention. Detailed information on patient characteristics and previous treatments can be found in Table 1. Patient-reported outcomes at baseline, 1-month follow-up and 3-month follow-up can be found in Table 2.

**Table 1.** Patient characteristics and previous treatments.

Case	Age	Sex	Smoking	Year of Crohn’s diagnosis	Treatment regimen at start of HBO	Previous medical treatment	Previous surgical treatment
1	54	Female	3 cigarettes a day	1976	None	Anti-tumour necrosis factor alpha treatment, methotrexate, prednisone, prolonged courses of antibiotics	Colectomy (1988), intersphincteric proctectomy (1992), excision sphincter complex (1992), mesorectal excision, omental and oblique rectus abdominis musculocutaneous flap (2017)
2	34	Female	No	2004	Mercaptopurin, antibiotics (stopped after start HBO)	Infliximab, azathioprine, adalimumab, vedolizumab, prolonged courses of antibiotics	Deviating transverso-stoma (2015), subtotal colectomy and ileostoma (2016, proctectomy and mesorectal excision with omental flap (2016)
3	33	Female	No	2000	None	Infliximab, adalimumab, certolizumab, ustekinumab azathioprine, mercaptopurine, cladribine, tiogunanine, purinethol, prednisolone, methotrexate, prolonged courses of antibiotics, granulocyte colony-stimulating factor/filgrastim	Ileostomy (2002), colectomy (2004), proctectomy (2013), mesorectal excision and omental flap (2018)

HBO: hyperbaric oxygen therapy.

**Table 2.** Patient-reported outcomes.

	EQ-VAS			IBDQ			FSFI		
	Pre	Post	FU	Pre	Post	FU	Pre	Post	FU
Case 1	90	80	80	210	208	204	5.4	24.5	6.0
Case 2	50	40	52	154	164	176	23.9	19.2	22.2
Case 3	30	44	46	125	138	155	5	19.7	24.6

EQ-VAS: EuroQol Visual Analogue Scale; IBDQ: Inflammatory Bowel Disease Questionnaire; FSFI: Female Sexual Function Index; FU: follow-up.

### Case 1

Case 1 had complaints of ongoing perineal wounds after a proctectomy in 1992, with excision of the mesorectum with omental flap and excision of an extensive perineal fistula complex with oblique rectus abdominis musculocutaneous flap in 2017 (2 years before the start of HBO). After this surgery, her perineal wounds improved, but she continued to have ulcerations in the perineal skin, with varying complaints of discharge and irritation. Magnetic resonance imaging (MRI) showed infiltration of the perineal area. At the start of HBO, small ulcerations in the gluteal cleft were present.

**Figure 1.**
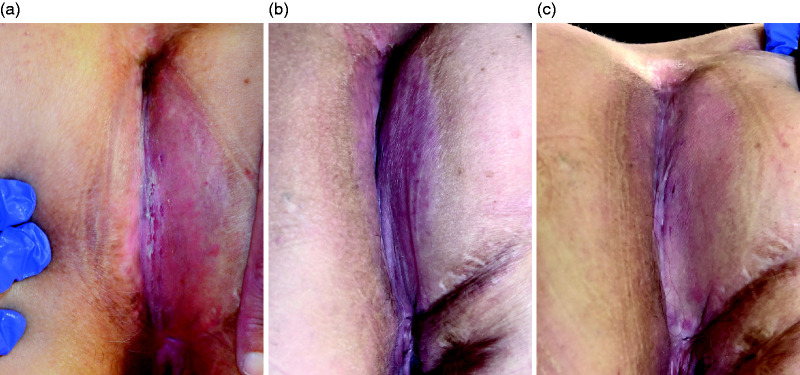
Photographs of case 1 before (a), 1 month after (b) and 3 months after (c) hyperbaric oxygen therapy (HBO).

**Figure 2.**
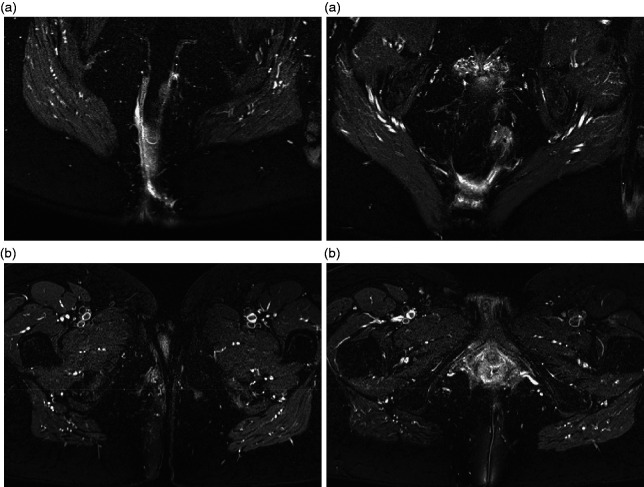
Magnetic resonance imaging of case 1 before (a) and 3 months after (b) HBO.

HBO was given without interruption and, other than fatigue as a solicited event, without side effects. One month after HBO, the perineal skin had completely healed (Figure 1). An MRI performed 2 months after HBO showed a reduction in infiltration (Figure 2). After 3 months of follow-up, the patient remained symptom free. The questionnaires showed a temporary increase in sexual functioning at 1-month follow-up; the IBDQ remained unchanged (the patient was already in remission at baseline).

### Case 2

Case 2 had her last major surgery (mesorectal excision and omental flap) 3 years before the start of HBO, with ongoing perineal wounds and abscesses ever since. An MRI performed a month before the start of therapy showed a perineal sinus tract with granulation tissue. Her treatment regimen at the start of HBO consisted of mercaptopurin and antibiotics (clindamycin) at maintenance dose; the antibiotics were stopped at the beginning of HBO.

**Figure 3.**
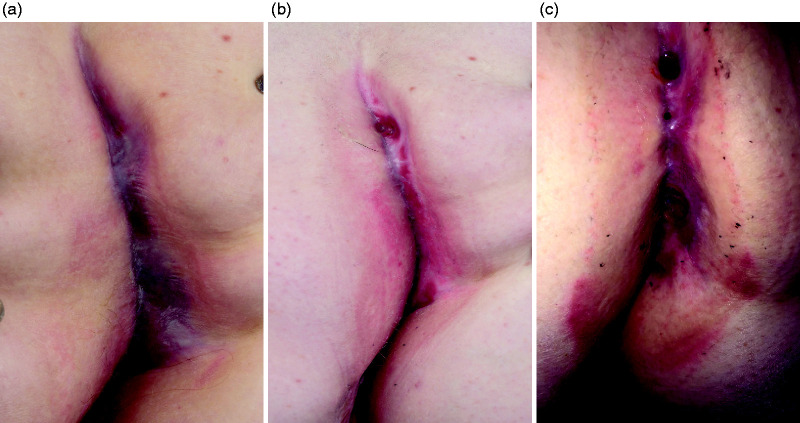
Photographs of case 2 before (a), 1 month after (b) and 3 months after (c) HBO.

The patient tolerated HBO well, with no side effects or interruptions of the regimen. During HBO, the lesions started producing more in the first few weeks, and the patient noticed an itching sensation in the wounds. At 1-month follow-up, the patient reported no significant change in complaints, with the wound showing a slight improvement (Figure 3).

Shortly hereafter, she reported more complaints of the perineal skin, and she also developed a new abscess in her axilla (likely to be related to the hidradenitis suppurativa). She was prescribed corticosteroid cream for the perineal wound, but complaints continued to increase. An MRI showed a significant deterioration of the sinus tract, with a new abscess of 2.5 cm × 1.0 cm and increased inflammation around the tract. Concomitantly, she developed a fistula next to her ileostoma, and mild luminal Crohn’s activity in the terminal ileum. She was started on ustekinumab at the end of follow-up. Contradictory, the IBDQ showed a meaningful improvement in complaints, with the patient being in remission at 3-month follow-up.

### Case 3

Case 3 had a proctectomy 4 years before the start of HBO, with a mesorectal excision and omental flap a year before HBO because of ongoing perineal complaints. Despite these treatments, she continued to have extensive perineal and vulvar skin defects that produced fluid and pus on a daily basis. Previously, all local and systemic (biological) therapies, including experimental filgrastim injections, had failed. Apart from pain medication, she was not using any concomitant medication for her complaints at the start of HBO.

During the course of HBO, she had one event of difficulty equalising, with otoscopy showing no barotrauma. Furthermore, a course of antibiotics was started after 10 sessions due to dysuria (which was a common complaint for her), unfortunately resulting in a high-output stoma and acute deterioration of kidney function caused by subsequent dehydration. She was treated with intravenous fluids, and kidney function returned to normal within one day, causing her to miss two sessions of HBO in total. This event was deemed unrelated to the HBO.

**Figure 4.**
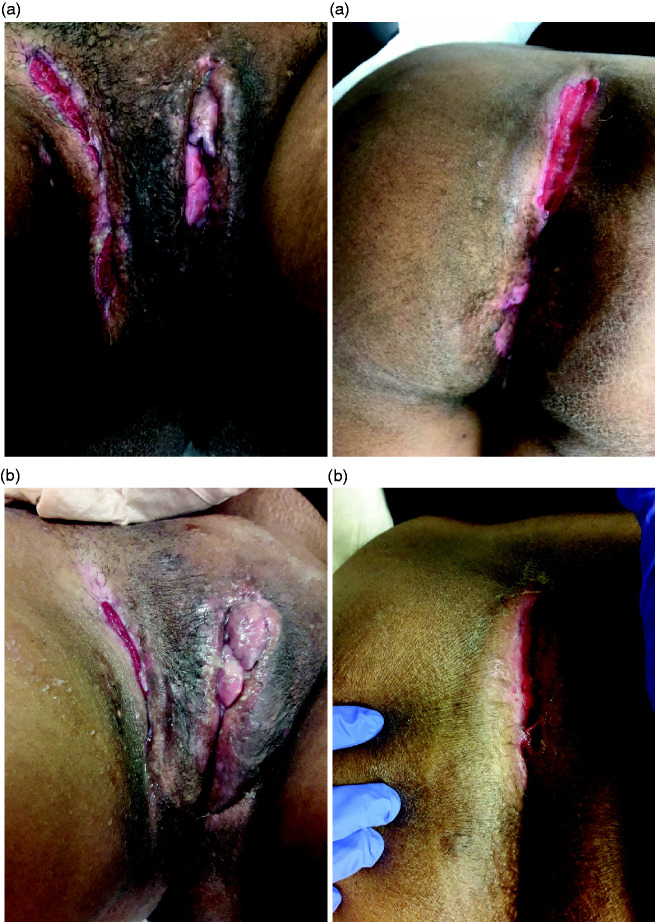
Photographs of case 3 before (a) and 1 month after (b) HBO.

During HBO, the patient experienced less production and pain of the inguinal and perineal wounds. However, her vulvar wounds caused more complaints, and she was seen by the dermatologist and gynaecologist after 25 sessions. A subscription for prednisone (10 days) was provided. Two weeks later, she reported less pain and irritation, which stayed the same for a few weeks in which she even had coitus successfully. One and a half months after HBO, she reported a temporary increase in production of the inguinal and perineal wounds. Her questionnaires showed improvement at both 1 and 3 months of follow-up (Table 2), with the IBDQ showing clinical remission and the FSFI a clear increase in sexual function. The photographs at the 1-month follow-up showed little changes compared with baseline (Figure 4). Unfortunately, she did not attend the 3-month follow-up visit, and no photographs are available for this time point.

## Discussion

In this article, the case histories of three patients with biopsy-proven metastatic perineal CD treated with HBO are presented. It showcases how difficult the management of this extra-intestinal manifestation can be, often requiring multiple (adjunctive) treatments, and how different the course of disease can be between patients. It also emphasises that metastatic CD does not necessarily mirror luminal activity. This therapy-refractory patient group had had all available treatment in the past, including mesorectal excision in order to remove all pro-inflammatory tissue, without sufficient response of the metastatic CD.^15^ There were also no indications of luminal activity elsewhere in the GI tract before the start of HBO. Furthermore, this research contributes to the clinical knowledge of the use of HBO as an adjunctive treatment in these patients.

Metastatic CD is generally considered a rare disease, being described in as few as 100 cases between 1965 (when it was first described) and the present.^3,4^ However, to our knowledge, data on the prevalence and/or incidence in CD patients is not available. The great variety in macroscopic appearance, the need for biopsy for a definite diagnosis and high sampling error for granulomas adds to the difficulty of establishing its occurrence.^4^

In this study, metastatic CD occurred in 46% of patients with ongoing wound-healing problems for more than 1 year after proctectomy. It is possible that the occurrence is even higher, since metastatic CD could not be ruled out in one patient due to cauterisation of the tissue, and three patients did not have biopsies. Even though this is a highly selected cohort (and these results cannot be extrapolated to all CD patients), it implies that metastatic CD might not be as rare as currently thought. All patients who had positive biopsies had perianal complaints before their proctectomy, with earlier research also indicating that preoperative perianal sepsis predicts poor healing of the perineal wound after proctectomy.^16^ It is possible that the granulomas were actually residual CD activity instead of metastatic CD. Regardless of the definition, given its implications for the management of non-healing wounds, (repeated) biopsy with a specific request to the pathologist to check for granulomas should be performed in patients experiencing non-healing after proctectomy.

HBO has been used as an (adjunctive) treatment in one earlier case report, concerning a 48-year-old woman with therapy-refractory metastatic CD of the perineum.^6^ After 8 years of frequent painful exacerbations requiring hospitalisation, she was treated with five courses of HBO, ranging from 18 to 67 sessions per course across a total of 2 years. She continued to have small perineal lesions, but compared with the 8 years prior to HBO, the extent of her perineal disease was deemed minimal, and she no longer required hospitalisation. The authors concluded that she was essentially asymptomatic after the treatment, and that HBO might have a possible role as an adjunct to other treatment regimens.^6^

The rationale for the use of HBO patients with metastatic CD is based on pathophysiological mechanisms of wound healing in general, as well as IBD-specific mechanisms. In general, the hyper-oxygenation and oxidative stress associated with HBO has been shown to result in vasoconstriction, decreasing local oedema, stem-cell mobilisation, fibroblast activation, up‐regulation of growth factors, antibacterial effects and potentiation of antibiotics, and a reduction in leucocyte chemotaxis.^9,17,18^ These effects have led to the use of HBO for established indications (such as diabetic foot ulcers), as well as experimental use in chronic non-healing wounds with other aetiologies such as calciphylaxis cutis or livedoid vasculopathy.^19,20^ Moreover, there are several anti-inflammatory properties of HBO that make it of special interest in the use of IBD. Animal models have shown decreased markers of inflammation, immune dysregulation and oxidative stress after HBO, as well as decreased oedema or colonic tissue weight and microscopic improvements on histopathological examination.^21^ Decreased levels of in pro-inflammatory cytokines (interleukin-1, interleukin-6 and tumour necrosis factor alpha) have also been confirmed in vivo in patients with CD.^7^ A recent randomised sham-controlled trial showed that a significantly higher proportion of patients with a moderate to severe flare of ulcerative colitis achieved clinical remission after HBO as an adjunct treatment on steroids than after sham treatment.^12^ Furthermore, a lower proportion of patients needed second-line rescue therapy or colectomy. Currently, a study concerning the use of HBO in patients with therapy-refractory perianal fistulising CD is being performed in the Amsterdam UMC as well.^22^

Parallel to the execution of this research, another patient with metastatic CD has been treated with HBO within the Amsterdam UMC. This was a female patient who had a colostomy (with the rectum still in situ) due to pancolitis, multiple perianal fistulas and large pararectal wounds with biopsy-proven metastatic CD requiring six surgical interventions in the year before HBO. She was treated with 40 sessions of HBO, after which no apparent clinical features of metastatic CD remained at the 3-month follow-up. She required no new surgical interventions, and a MRI showed reduction of the fistula complex, allowing her to be scheduled for surgical closure of the fistula.

The pathophysiological mechanisms, positive effects in IBD in earlier clinical trials and results seen in this case series imply that HBO might have a place in the medical management of patients with metastatic CD. The treatment was well tolerated in all patients. It is unclear what amount of sessions is optimal for these patients, and if a long-lasting improvement can be expected. Expanding the case series with more patients will help to establish better the position and timing of hyperbaric treatment in the treatment of metastatic CD. Given the low number of patients reported to have metastatic CD to date, and the broad range of patient characteristics, performing large and/or controlled trials to establish a causal effect of HBO is not realistic.
